# Role of Protein Kinase C, PI3-kinase and Tyrosine Kinase in Activation of MAP Kinase by Glucose and Agonists of G-protein Coupled Receptors in INS-1 Cells

**DOI:** 10.1155/EDR.2001.233

**Published:** 2001

**Authors:** Dietmar Böcker, Eugen J. Verspohl

**Affiliations:** 1 Department of Pharmacology Institute of Pharmaceutical Chemistry University of Münster Hittorfstr. 58-62 Münster 48149 Germany

## Abstract

MAP (mitogen-activated protein) kinase (also called Erk 1/2) plays a crucial role in cell proliferation and differentiation. Its impact on secretory events is less well established. The interplay of protein kinase C (PKC), PI3-kinase nd cellular tyrosine kinase with MAP kinase activity using inhibitors and compounds such as glucose, phorbol 12-myristate 13-acetate (PMA) and agonists of G-protein coupled receptors like gastrin releasing peptide (GRP), oxytocin (OT) and glucose-dependent insulinotropic peptide (GIP) was investigated in INS-1 cells, an insulin secreting cell line. MAP kinase activity was determined by using a peptide derived from the EGF receptor as a MAP kinase substrate and [P32]ATP. Glucose as well as GRP, OT and GIP exhibited a time-dependent increase in MAP kinase activity with a maximum at time point 2.5 min. All further experiments were performed using 2.5 min incubations. The flavone PD 098059 is known to bind to the inactive forms of MEK1 (MAPK/ERK-Kinase) thus preventing activation by upstream activators. 20 μM PD 098059 (${\text{IC}}_{50}$=51 μM) inhibited MAP kinase stimulated by either glucose, GRP, OT, GIP or PMA. Inhibiton (“downregulation”) of PKC by a long term (22h) pretreatment with 1 μM PMA did not influence MAP kinase activity when augmented by either of the above mentioned compound. To investigate whether PI3-kinase and cellular tyrosine kinase are involved in G-protein mediated effects on MAP kinase, inhibitors were used: 100 nM wortmannin (PI3-kinase inhibitor) reduced the effects of GRP, OT and GIP but not that of PMA; 100 μM genistein (tyrosine kinase inhibitor) inhibited the stimulatory effect of either above mentioned compound on MAP kinase activation. Inhibition of MAP kinase by 20 μM PD 098059 did not influence insulin secretion modulated by either compound (glucose, GRP, OT or GIP). [H3]Thymidine incorporation, however, was severely inhibited by PD 098059. Thus MAP kinase is important for INS-1 cell proliferation but not for its insulin secretory response with respect to major initiators and modulators of insulin release. The data indicate that MAP kinase is active and under the control of MAP kinase. PKC is upstream of a genisteinsensitive tyrosine kinase and probably downstream of a PI3-kinase in INS-1 cells.


*Int. Jnl. Experimental Diab. Res.*, Vol. 2, pp. 233–244
Reprints available directly from the publisher
Photocopying permitted by license only

© 2001 OPA (Overseas Publishers Association) N.V.
Published by license under
the Harwood Academic Publishers imprint,
part of Gordon and Breach Publishing,
a member of the Taylor & Francis Group.
Printed in the U.S.A.

### Role of Protein Kinase C, PI3-kinase and Tyrosine Kinase in Activation of MAP Kinase by Glucose and Agonists of G-protein Coupled Receptors in INS-1 Cells

DIETMAR BÖCKER and EUGEN J. VERSPOHL*

*Department of Pharmacology, Institute of Pharmaceutical Chemistry, University of Münster, Hittorfstr. 58-62, 48149 Münster, Germany*

*(Received 3 May 2001; In final form 21 August 2001)*

**MAP (mitogen-activated protein) kinase (also called Erk 1/2) plays a crucial role in cell proliferation and differentiation. Its impact on secretory events is less well established. The interplay of protein kinase C (PKC), PI3-kinase and cellular tyrosine kinase with MAP kinase activity using inhibitors and compounds such as glucose, phorbol 12-myristate 13-acetate (PMA) and agonists of G-protein coupled receptors like gastrin releasing peptide (GRP), oxytocin (OT) and glucose-dependent insulinotropic peptide (GIP) was investigated in INS-1 cells, an insulin secreting cell line. MAP kinase activity was determined by using a peptide derived from the EGF receptor as a MAP kinase substrate and [$^{32}$P]ATP. Glucose as well as GRP, OT and GIP exhibited a time-dependent increase in MAP kinase activity with a maximum at time point 2.5 min. All further experiments were performed using 2.5 min incubations. The flavone PD 098059 is known to bind to the inactive forms of MEK1 (MAPK/ERK-Kinase) thus preventing activation by upstream activators. 20 μM PD 098059 ($IC_{50}$ = 5 μM) inhibited MAP kinase stimulated by either glucose, GRP, OT, GIP or PMA. Inhibiton ("downregulation") of PKC by a long term (22 h) pretreatment with 1 μM PMA did not influence MAP kinase activity when augmented by either of the above mentioned compound. To investigate whether PI3-kinase and cellular tyrosine kinase are involved in G-protein mediated effects on MAP kinase, inhibitors were used: 100 nM wortmannin (PI3-kinase inhibitor) reduced the effects of GRP, OT and GIP but not that of PMA; 100 μM genistein (tyrosine kinase inhibitor) inhibited the stimulatory effect of either above mentioned compound on MAP kinase activation. Inhibition of MAP kinase by 20 μM PD 098059 did not influence insulin secretion modulated by either compound (glucose, GRP, OT or GIP). [$^3$H]Thymidine incorporation, however, was severely inhibited by PD 098059. Thus MAP kinase is important for INS-1 cell proliferation but not for its insulin secretory response with respect to major initiators and modulators of insulin release. The data indicate that MAP kinase is active and under the control of MAP kinase. PKC is upstream of a genistein-sensitive tyrosine kinase and probably downstream of a PI3-kinase in INS-1 cells.**

*Keywords*: MAP kinase; INS-1 cells; Insulin release; Cell proliferation

### INTRODUCTION

MAP kinases belong to the family of serin/threonin phosphorylating kinases. Two isoenzymes of MAP kinase have been detected by immunoprecipitation[1] and by Western blotting.[2] Its activation results in mitosis, meiosis, cell differentiation and development. Though it is well known that receptors for peptidergic growth factors with

*Address for correspondence: Department of Pharmacology, Institute of Pharmaceutical Sciences, Hittorfstr. 58-62, 48149 Münster, Germany. e-mail: verspoh@uni-muenster.de intrinsic tyrosine kinase activity are involved in cell proliferation *via* MAP kinases, it is not clear whether G-protein coupled receptors are involved in proliferation in addition to their functions in fully differentiated cells.[3,4] GRP (gastrin releasing peptide), OT (oxytocin) and GIP (glucose-dependent insulinotropic peptide) were chosen for our experiments. All compounds were selected due to the fact that their effects are mediated *via* G-proteins: The 2nd messengers are PLC (for GRP and OT; probably coupled to $G_q$) and mainly adenylylcyclase (for GIP; probably coupled to $G_s$). These compounds are modulators of insulin release.[5–7] Using these compounds and specific inhibitors it is interesting to know whether MAP kinase is involved in insulin release and important for cell proliferation of INS-1 cells since there is a dynamic replicaton of the $\beta$-cell mass[8] and the production of new $\beta$-cells is approx 3% per day in adult rats and mice.[9]

The MAP cascade may be activated by receptor tyrosine kinases, receptors for cytokines and G-protein coupled receptors. MAP kinase (ERK 1/2 (extracellularly regulated kinase)) is stimulated by MAPK kinase/MEK 1/2. Many rather specific compounds help to find out the importance and regulation of the MAP kinase cascade: PD 098059 is able to inhibit the cascade on the level of MAP kinase,[10,11] wortmannin as a PI3-kinase inhibitor is able to define the involvement of the enzyme in the cascade activity, genistein (a tyrosine kinase inhibitor) may be used to investigate the influence of tyrosine kinase on MAP kinase as originally described by Hordijk *et al.*[12] Tyrosine kinases may be involved in the activation of MAP kinase by G-protein coupled pathways.[12] Depending on the investigated tissue PKC is involved in the G-protein mediated activation of MAP kinase.[13–17]

Except the studies of Frödin *et al.*[1] no specific investigation on MAP kinase in insulin secreting cells with respect to G-protein coupled receptors exist. The aim of the present study was to answer the question whether MAP kinase is involved in mediating insulin secretion and/or cell proliferation of INS-1 cells and whether the signal transduction pathway (MAP kinase cascade) is involved in effects of glucose, GRP, oxytocin, GIP (G-Protein coupled receptor agonists) and the phorbolester PMA. The cross-talk within the cells with respect to tyrosine kinase, protein kinase C (PKC) and PI3-kinase was investigated as well in order to look at upstream and downstream cascades.

### MATERIALS AND METHODS

### Materials

Bovine serum albumin, genistein, oxytocin, IGF-1, PMA, tyrphostin AG 1296, wortmannin, SBTI (soybean trypsin inhibitor) and bacitracin were obtained from Sigma-Aldrich (Deisenhofen, Germany) and collagenase (CLS grade, 126–196 U/ml) from Worthington Biochemicals Corp. (Freehold, New Jersey). Porcine gastrin releasing peptide$_{1\text{-}27}$ (GRP(1-27)) was purchased from Bachem Biochemica GmbH, Heidelberg, Germany. GIP (glucose-dependent insulinotropic peptide) were purchased from Calbiochem-Novabiochem GmbH, Bad Soden/Ts, Germany, (mono-$^{125}$I-Tyr$^{A14}$)-porcine insulin (360 mCi/mg) from Behringwerke AG (Marburg, Germany), rat insulin from Novo Research Institute (Copenhagen, Denmark), and anti-insulin antibodies from Linco (St. Louis, U.S.A.). Biotrak MAP Kinase assay kit, [$\gamma$-$^{32}$P]ATP, [methyl-$^{3}$H]thymidine, Q-Sepharose Fast Flow were purchased from Amersham Pharmacia Biotech (Freiburg, Germany), PD 098059 from New England Biolabs (Schwalbach/Ts. Germany) and pertussis toxin from Calbiochem-Novabiochem GmbH (Bad Soden/Ts., Germany).

### INS-1 Cell Culture

INS-1 cells[18] generously provided by Dr. C. Wollheim (Geneva, Switzerland) were plated at a density of $5 \times 10^5$ cells/ml and grown in RPMI 1640 medium supplemented with 10% (v/v) fetal bovine serum, 10 mM HEPES, 2 mM glutamine, 1 mM pyruvate, 50 μM mercaptoethanol, 100 U/mL penicillin and 0.1 mg/mL streptomycin.

### Animals

Adult Wistar rats of either sex weighing between 230 and 330 g were used. They were kept on a standard pellet diet (Altromin, Lage) and tap water ad libitum at 22°C with a 12 h light/dark rhythm.

### Isolation of Rat Pancreatic Islets

Isolation of pancreatic islets was as described by Kuo *et al.*[19] with slight modifications.[20] Pancreata were isolated from the ether-anesthetised rat, minced, and washed twice with 20 ml ice-cold KRH (Krebs-Ringer-Hepes) solution containing 2.8 mM glucose, 1 mg/ml bacitracin, 0.2 mg/ml SBTI, and 0.02% albumin. Pancreas pieces were soaked and then shaken in a 37°C water bath in the presence of 650 U collagenase/g tissue suspension. After 15–18 min of incubation the tissue suspension was transferred into 10 ml of ice-cold KRH buffer. Islets were separated by sedimentation and collected as described elsewhere.[19]

### Insulin Secretion and Radioimmunoassay

INS1 cells were plated at $1{,}5 \times 10^5$ cells/well in 24-well plates and cultured for 5 days. Cells were washed three times with Krebs-Ringer buffer containing 10 mM HEPES and 0.5% bovine serum albumin (KRH-buffer). The cells were preincubated with KRH-buffer containing 3.0 mM glucose in the presence or absence of the respective inhibitor for 30 minutes and incubated at 37°C in KRH-buffer containing 8.3 mM glucose and the test compound. 5 pancreatic islets were incubated for 90 min at 37°C in KRH buffer containing either 3.0 or 11.1 mM glucose with or without PD 098059.

Insulin release into the medium was determined by a radioimmunoassay using rat insulin as a standard, (mono-$^{125}$I-Tyr$^{A14}$)-porcine insulin as the labeled compound and anti-insulin antibodies. The intraassay and interassay variabilities were 4.2 and 9.8% respectively. Each compound had been checked for non-interference with the insulin radioimmunoassay.

### [$^3$H]Thymidine Incorporation

INS-1 cells in 24 well plates were grown for 3 days until being subconfluent. From the 4th day they were incubated for 72 hours with the test compounds in a serum free medium containing 0.1% BSA instead of fetal calf serum, transferrin (10 μg/ml), triiodthyronin (0.1 nM), phosphoethanolamine (50 μM), ethanolamine (50 μM) and IGF-I (0.65 nM). In case the MEK inhibitor PD 098059 was investigated, the cells were preincubated for 30 min in its presence. During the last 24 hours of incubation 0.5 μCi [$^3$H]thymidine was added per well. Thereafter cells were transferred on ice, sucked from medium, and 1 ml of methanol was added for 10 min (increase of cell attachment). Cells were rinsed twice with KRH buffer and with ice-cold 0.3 N TCA. Cells were lysed with 250 μl 0.3 N NaOH (30 min 37°C). Radioactivity (incorporated into DNA) present in samples of the extracts was measured using a scintillation counter.

### Micro-trap Phosphorylation Assay (Map Kinase Activity)

The micro-trap assay was conducted as described previously with slight modifications.[21] $2.6 \times 10^4$ cells per well were grown for 4 or 5 days in 96-well microtiter plates. The normal incubation medium was replaced by a serum free medium containing 0.1% BSA instead of fetal calf serum. After another 24 hours cells were rinsed twice followed by a preincubation with KRH containing 1 mM glucose. In case the MEK inhibitor PD 098059 was investigated, the cells were preincubated for 30 min in its presence. Main incubation was for 2.5 to 30 min as indicated in the legends. To finish incubation wells were transferred on ice, incubation media were discarded and cells were frozen at −70°C for 30 min. The frozen cells were lysed in 100 μl of ice-cold lysis buffer (20 mM Tris-HCl, pH 7.5, 1 mM $Na_3VO_4$, 25 mM glycerophosphate, 2 mM EGTA, 1 mM PMSF, 20 μg/ml aprotinin, 2 mM DTT, and 100 mM NaCl, and then transferred to multiscreen plate (MADVN6510, Millipore,

Eschborn, Germany). This lysate was incubated 1 : 1 with 50% (v/v) slurry of Q-Sepharose/lysis buffer and was shaken on a microplate mixer at 4°C for 30 min. This plate was set onto the multiscreen-filtration holder (Millipore) with a suction pump. The lysis buffer was aspirated and the Q-Sepharose beads were washed twice with fresh lysis buffer. Then 100 μl of the elution buffer (identical to lysis buffer except containing 300 mM of NaCl instead of 100 mM) was added, and the eluted sample was collected by aspiration in a new plate set under the filtration holder. The partially purified MAP kinase was used to determine its activity by mixing 15 μl of a sample with 10 μl substrate buffer with a peptide that contains a partial phosphorylation domain of the EGF receptor (Amersham); Hepes, $Na_3VO_4$, 0.05% $NaN_3$, pH 7.4 and 5 μl (1 μCi) Mg-[$^{32}$P]ATP which is well mixed. After a 30 min incubation at 37°C, the reaction was terminated by adding 15 μl of phosphoric acid. Then 30 μl of each sample were spotted onto special binding discs (Biotrak MAP kinase assay kit). After extensive washing twice for 2 min with 5% phosphoric acid and twice with water, the discs were counted in scintillation counter.

### Statistics

Results are shown as means ± S.E.M. Statistical significance was determined using one-way analysis of variance (ANOVA) (RS/1 statistics pack, BBN Software Products Corp.) followed by a post-hoc test (Newman Keuls). A p value $< 0.05$ was considered significant.

### RESULTS

### Map Kinase Activity

As shown in Figures 1A and B glucose (an initiator of insulin release) as well as GRP, OT and GIP (modulators of insulin release) exhibit a distinct time profile with respect to activation of MAP kinase activity: There is a maximum after 2.5 min. The activity comes down shortly, and there is a 2nd peak after 10 min which fades away slowly. Either modulator tested superimposes the maximum observed for glucose alone after 2.5 min (458, 373 and 386% compared with 292%) (Fig. 1B). Our data on glucose corroborate those found by others.[1,2] All further experiments on MAP kinase were performed after a 2.5 min incubation. Since the compounds are modulators of insulin release they were always used in combination with glucose.

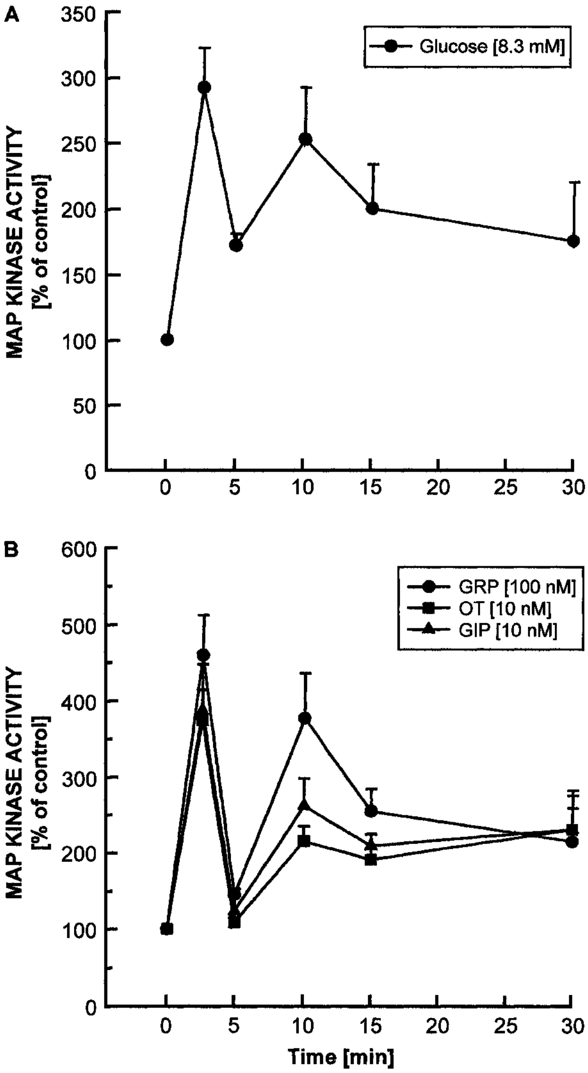

FIGURE 1 Time-course of various compounds on MAP kinase activity. A: glucose effect (8.3 mM); B: effects of GRP, OT and GIP. The activity of INS-1 cells during preincubation time was 100%. Results are shown as mean ± S.E.M. of 3–14 independent experiments run in quadruplicates.

Next the effect of the MEK inhibitor, PD 098059,[11] was investigated. The half-maximal concentration for inhibiting MAP kinase activity in INS 1 cells was approx. 5 μM when either glucose or a combination of glucose plus GRP was used (data not shown). Experiments with PD 098059, therefore, were performed at 20 μM concentrations. 20 μM PD 098059 was able to inhibit MAP kinase activity when stimulated by either high glucose (8.3 mM) or GRP, OT, GIP or the phorbol ester PMA (Fig. 2). Glucose at a low concentration (3.0 mM) had a small effect (Fig. 2). The effect of PMA has already been shown by others in a similar way.[1]

Pertussis toxin at a concentration of 100 ng/ml had no influence on MAP kinase activity (data not shown).

To investigate the downstream and upstream cascade of MAP kinase various inhibitors of tyrosine kinase, PI3-kinase and PKC were used. Next the effect of tyrosine kinase inhibitors on MAP kinase was investigated. Genistein is known to inhibit cytosolic and receptor linked tyrosine kinases.[22] A concentration of 100 μM for genistein was used which was optimal and non-toxic in insulin releasing experiments.[23] 100 μM genistein significantly inhibited the stimulatory effects of glucose (8.3 mM), GRP, OT, GIP and PMA (Fig. 3A). AG 1296 is a tyrosine kinase inhibitor from the tyrphostin family which rather selectively inhibits the kinase of the PDGF (platelet derived growth factor) receptor. AG 1296 has no effect on MAP kinase activity

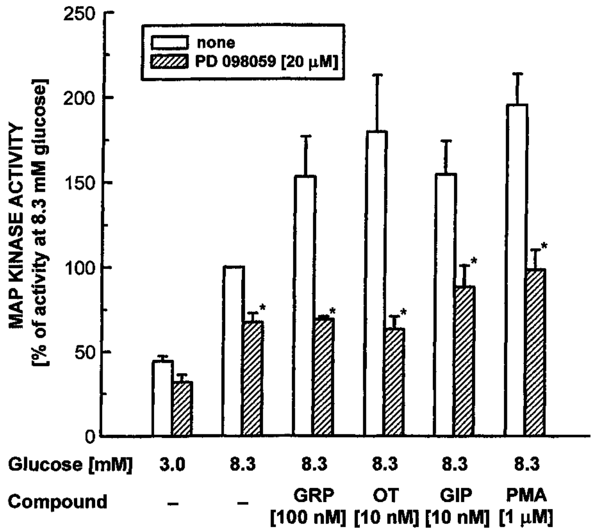

FIGURE 2 Effect of PD 098059 on MAP kinase activity stimulated by either GRP, OT, GIP or PMA. INS-1 cells were preincubated with 20 μM PD 098059 at 3.0 mM glucose, and then incubated for 2.5 min in the presence of either compound. Results are shown as mean ± S.E.M. of 3–6 independent experiments run in quadruplicates. Data were normalized to 8.3 mM glucose in the absence of PD 098059 (controls). *$p < 0.05$ *vs.* absence of PD 098059.

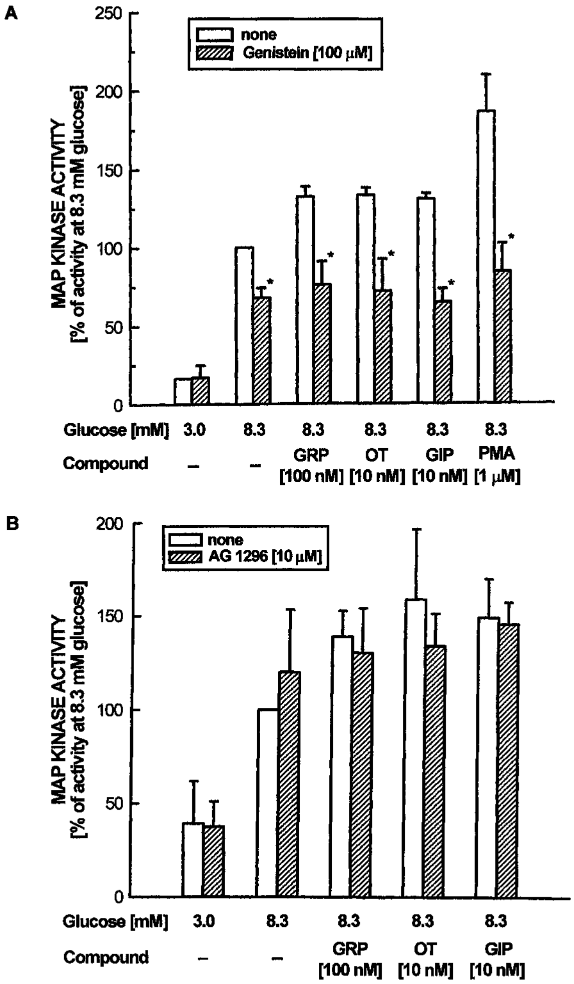

FIGURE 3 Effect of genistein and AG 1296 on MAP kinase activity stimulated by either GRP, OT, GIP or PMA. INS-1 cells were preincubated at 3.0 mM glucose with either 100 μM genistein for 30 min (A) or 10 μM AG1296 for 60 min (B); then the cells were incubated for 2.5 min in the presence of either indicated compound. Results are shown as mean ± S.E.M. of 3–7 independent experiments run in quadruplicates. Data were normalized to 8.3 mM glucose in the absence of genistein or AG 1296. *$p < 0.05$ *vs.* absence of genistein.

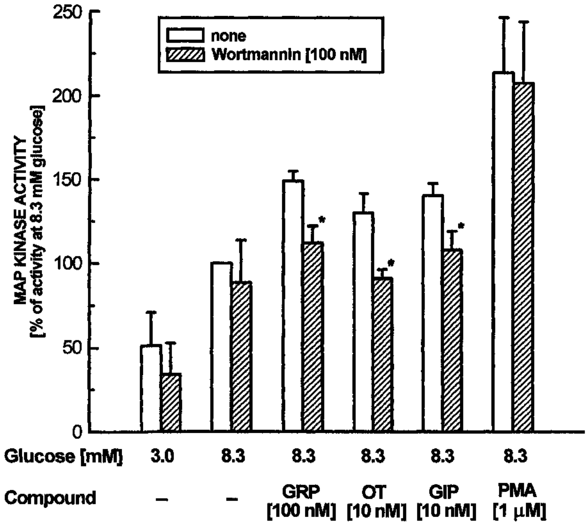

FIGURE 4 Effect of wortmannin on MAP kinase activity stimulated by either GRP, OT, GIP or PMA. INS-1 cells were preincubated with 100 nM wortmannin at 3.0 mM glucose for 30 min, and then incubated for 2.5 min in the presence of either compound. Results are shown as mean ± S.E.M. of 3–5 independent experiments run in quadruplicates. Data were normalized to 8.3 mM glucose in the absence of wortmannin. *$p < 0.05$ *vs.* absence of wortmannin.

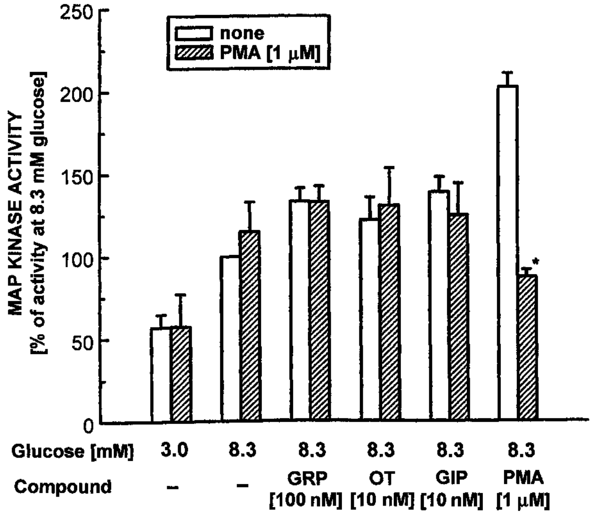

FIGURE 5 Effect of PMA-pretreatment on MAP kinase activity stimulated by either GRP, OT, GIP or PMA. INS-1 cells were preincubated with 1 μM PMA at 3.0 mM glucose for 22 hours, and then incubated for 2.5 min in the presence of either compound. Results are shown as mean ± S.E.M. of 3–5 independent experiments run in quadruplicates. Data were normalized to 8.3 mM glucose in the absence of PMA-pretreatment. *$p < 0.05$ *vs.* absence of PMA-pretreatment.

(Fig. 3B) indicating that a transactivated PDGF tyrosine kinase is not involved.

The effect of the PI3-kinase inhibitor wortmannin was investigated. 100 nM wortmannin are able to inhibit the stimulatory effect of GRP, OT and GIP on MAP kinase (Fig. 4). The effects of both the phorbolester PMA and of glucose at two different concentrations are not affected by wortmannin (Fig. 4).

As shown in Figure 5 1 mM PMA has an acutely stimulating effect on MAP kinase which is abolished by a 22-hours pretreatment with 1 mM PMA. PMA, however, is not able to inhibit the stimulatory effect of other compounds on MAP kinase such as glucose, GRP, OT or GIP (Fig. 5).

### Biological Effects (Insulin Secretion and Cell Proliferation)

It is interesting to know whether MAP kinase activity is necessary for specific biological effects. Insulin release is augmented by those concentrations of glucose, GRP, OT and GIP which are able to increase MAP kinase activity (Fig. 6). The MEK inhibitor PD 098059, however, is not able to inhibit insulin release induced by either of the compounds (Fig. 6).

Cell proliferation was measured as [$^3$H]thymidine incorporation. Glucose and IGF-1 (as a control compound) increase cell proliferation. The other compounds investigated (GRP and GIP) had a tendency (not statistically significant) to increase [$^3$H]thymidine incorporation. This increase is inhibited by PD 098059 no matter the compound investigated (8.3 mM glucose, nM concentrations of GRP, OT, GIP and IGF-I) (Fig. 6B). This is also the case when a substimulatory glucose concentration (3.0 mM) was used (Fig. 6C) which by itself has a small proliferative effect (Fig. 6B); therefore the effects are not clearly glucose-dependent.

Since INS-1 cells have the characteristics of tumour cells and MAP kinases may play an important role for them to stay immortalized some data were repeated for freshly isolated rat pancreatic islets. As can be seen in Figure 7 20 μM

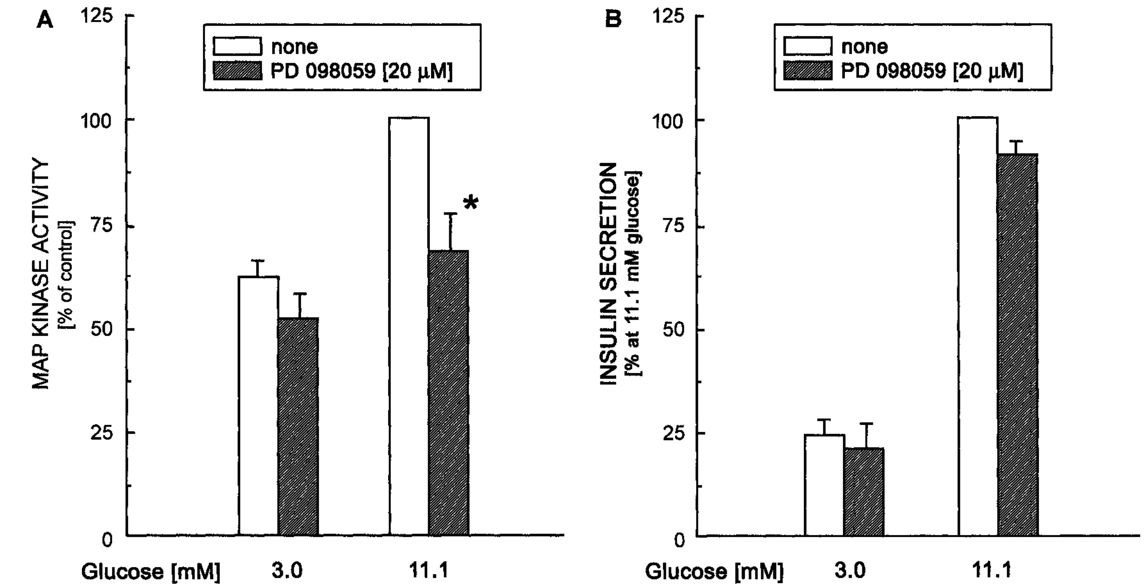

FIGURE 7 Effect of PD 098059 on MAP kinase activity (A) and glucose-induced insulin secretion (B) in rat pancreatic islets. Rat pancreatic islets were preincubated with 20 μM PD 098059 in KRH-buffer in the presence of 3.0 mM glucose for 30 min. Then the islets were incubated with 20 μM PD 098059 for 2.5 min (A: MAP kinase) or 90 min (B: insulin release) in the presence of either 3.0 or 11.1 mM glucose. Results are shown as mean ± S.E.M. of 4 independent experiments run in triplicates. *$p < 0.05$ *vs*. absence of PD 098059.

PD 098059 inhibit MAP kinase activity though not being able to inhibit insulin release under the conditions used.

### DISCUSSION

### Map Kinase

GRP, oxytocin and GIP activate MAP kinase in the presence of a stimulatory glucose concentration. The time-course is the same for glucose alone and its combination with the above mentioned compounds. The effects of GRP, oxytocin and GIP are specific since the MEK inhibitor PD 098059 is effective in inhibiting the MAP kinase activity probably by inhibiting the cascade upstream of MAP kinase.

Our data are in line with data in other cell systems: GRP is known to activate MAP kinase in Swiss-3T3 fibroblasts[24,25] and the maximum effect is also demonstrated after 2 min. Both OT and GIP have already been shown to activate MAP kinase in rat myometrial cells[26,27] and CHO cells,[28] respectively.

Stimulating MAP kinase shows a biphasic pattern with two distinct maxima: after 2.5 min and 10 min. Whereas the same time profile was found by some authors[29,30] but not by others.[1,2] This difference may be due to the fact that only the isoenzyme ERK1 was immunoprecipitated whereas in our and other studies both isoenzymes were estimated *e.g.*, by the microtrap assay. Another reason for the biphasic profile may be that MAP kinase phosphatase I[31] dephosphorylates MAP kinase 15 to 200 times more quickly than other tyrosine phosphorylated substrates which is severely active especially in the initial phase. An alternative explanation could be that we deal with a complex cascade which includes Shc, Grb2, Sos and Ras; since Sos can be phosphorylated by MAP kinase[32] it is possible that phosphorylated Sos induces a negative feedback in the cascade Ras/RAF/MEK/MAK kinase. A quick dephosphorylation of Sos may terminate the short negative feedback on MAP kinase.

Various tyrosine kinases have been shown to specifically interact with the cascade between

G-protein coupled receptors and MAP kinases.[33–37] In INS-1 cells the tyrosine kinase inhibitor genistein inhibits MAP kinase activity when stimulated by either glucose or combinations of glucose with GRP, oxytocin, GIP or PMA. Interestingly vasopressin possibly *via* $V_{1b}$ receptors stimulates a genistein-sensitive tyrosine kinase.[38]

PI3-kinase is involved in the G-protein mediated signal transduction which leads to activation of MAP kinase since the PI3-kinase inhibitor wortmannin inhibited MAP kinase activity when induced by glucose, GRP, oxytocin and GIP except PMA. PI3-kinase, however, is not involved in mediating insulin release[39,40] which hints already at a dissociation between MAP kinase and insulin secretion as discussed later. Extremely high concentrations of wortmannin (1 μM) are able to inhibit insulin release[41] which lead to nonspecific effects such as inhibition of phospholipase D; these high concentrations were not used in our experiments. Obviously PI3-kinase is part of the MAK kinase cascade when activated by GRP, OT or GIP confirming data shown for CHO fibroblasts.[42]

Activation of PKC is involved in the effects of GRP.[41] The oxytocin/vasopressin effect on MAP kinase is at least partially dependent on PKC.[38,43–46] Downregulation of the PMA-sensitive PKC isoforms by PMA does not influence MAP kinase activity stimulated by glucose, GRP, oxytocin and GIP. Only the acute PMA effect is inhibited (positive control experiment). No PMA-sensitive PKC isoforms, therefore, are involved in the effect of either compound with respect to MAP kinase activity. This is different with respect to PKC$\xi$, an atypical PKC isoenzyme, which is known to activate both MEK and MAP kinase.[47,48]

### Insulin Secretion

It is not clear whether MAP kinase is linked to insulin release. In our hands insulin secretion does not appear to be dependent on MAP kinase activity since manoeuvres to inhibit its activity, *e.g.*, by the MEK inhibitor PD 098059, does not result in changes in insulin release. This is in contrast to data of Frödin *et al.*:[1] They demonstrated concentration-response curves being parallel for the effect of glucose on MAP kinase activity and insulin release which, however, does not automatically implicate a causal link. In contrast using nerve growth factor there was no parallel effect[1] indicating that stimulation of MAP kinase alone does not result in an increase in insulin release.

### Cell Proliferation

Glucose is a major stimulus for cell proliferation of INS-1 cells as measured by [$^3$H]thymidine incorporation into cells corroborating data of others.[8,49,50] Cell proliferation was modulated by either compound in a tentative way (approx. 9%) but was not statistically significant. IGF-I (positive control) was effective but its effect was small compared to what was shown by others.[51] The lack of effect of oxytocin may be due to low expression of its receptors, since oxytocin had no effect on [$^3$H]thymidine incorporation in CHO cells[44] unless the cells had been transfected with cDNA of the vasopressin$_{1b}$ receptor. Our data with GRP are in line with the observation that GRP has proliferative effects in normal cells[52,53] but not in carcinoma cells.[54]

[$^3$H]Thymidine incorporation was inhibited by the MEK inhibitor PD 098059 at 8.3 mM glucose. Since the inhibition come down to the same level independent of the compound used, it is obvious that the glucose effect is inhibited. Additionally the proliferative effects of GIP and IGF-1 are inhibited even at low glucose (3.0 mM). Since GIP is an important incretin its proliferative effect may be of clinical importance. Altogether the MEK inhibitor is effective in blocking glucose effects; when proliferation is stimulated by high glucose concentrations, the additional small effects of GRP, GIP or IGF-I cannot be blocked. The proliferative effect of these compound is only blocked when low glucose concentrations (3.0 mM) were used.

A scheme illustrating the points that are addressed in this paper is shown in Figure 8.

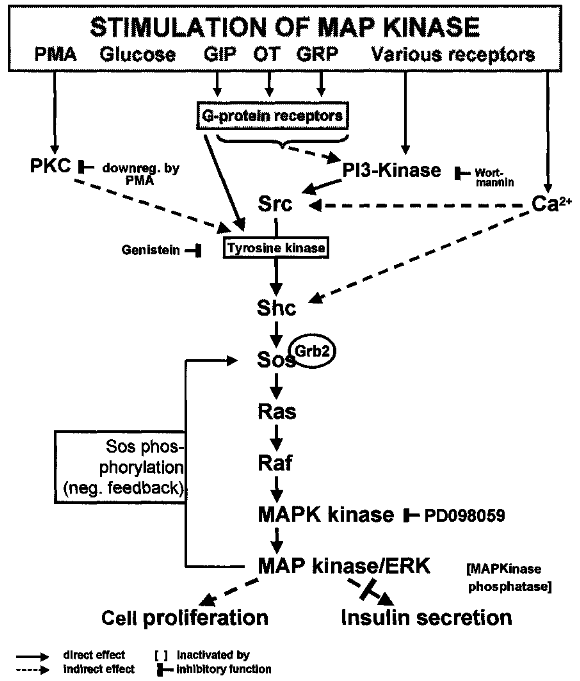

FIGURE 8 Schematic illustration of MAP kinase cascade with drugs and inhibitors used in the paper. (PMA = phorbol myristyl acetate, PKC = protein kinase C, Src = a protein tyrosine kinase, Shc = an adapter protein, Sos = son of sevenless, Grb2 = growth factor receptor-bound protein 2, Ras = protein family, oncogene, Raf = protein family, oncogene, ERK = extracellular signal regulated protein kinase, MAP kinase = mitogen-activated protein kinase, MAPK kinase = MAP kinase kinase).

With respect to freshly isolated rat pancreatic islets data on the effect of PD 098059 on both MAP kinase activity and insulin release at two different glucose concentrations were repeated showing no major difference to the INS-1 cells. With respect to glucose and PMA Persaud *et al.*[55] clearly showed that MAP kinase is not important for the insulin secretory process. Thus, not only cells being immortalized and therefore dependent on MAP kinase show that MAP kinase is not important for insulin release.

### CONCLUSION

1. MAP kinase is active in INS-1 cells.
2. MAP kinase is of no major importance for insulin release, but strongly affects cell proliferation with respect to various compounds such as glucose and GIP.
3. MAP kinase is activated after interaction of compounds with G-protein coupled receptors.
4. Glucose is the major stimulus for cell proliferation.
5. In the MAP kinase pathway PKC is upstream of a genistein-sensitive tyrosine kinase.
6. PKC is downstream of the PI3-kinase or alternatively there exist fully different signal transduction pathways for PKC and PI3-kinase.

### *References*

[1] Frödin, M., Sekine, N., Roche, E., Filloux, C., Prentki, M., Wollheim, C. B. and Van Obberghen, E. (1995). Glucose, other secretagogues, and nerve growth factor stimulate mitogen-activated protein kinase in the insulin-secreting beta-cell line, *J. Biol. Chem.*, **270**, 7882–7889.
[2] Khoo, S. and Cobb, M. H. (1997). Activation of mitogen-activating protein kinase by glucose is not required for insulin secretion, *Proc. Natl. Acad. Sci. USA*, **94**, 5599–5604.
[3] Yarden, Y., Escobedo, J. A., Kuang, W. J., Yang-Feng, T. L., Daniel, T. O., Tremble, P. M., Chen, E. Y., Ando, M. E., Harkins, R. N., Francke, U. *et al.* (1986). Structure of the receptor for platelet-derived growth factor helps define a family of closely related growth factor receptors, *Nature*, **323**, 226–232.
[4] Dohlmann, H. G., Caron, M. G. and Lefkowitz, R. J. (1987). A family of receptors coupled to guanine nucleotide regulatory proteins, *Biochemistry*, **26**, 2657–2664.
[5] Petterson, M. and Ahrén, B. (1987). Gastrin releasing peptide (GRP): effects on basal and stimulated insulin and glucagon secretion in the mouse, *Peptides*, **8**, 55–60.
[6] Bobbioni-Harsch, E., Frütiger, S., Hughes, G., Panico, M., Etienne, A., Zappacosta, F., Morris, H. R. and Jeanrenaud, B. (1995). Physiological concentrations of oxytocin powerfully stimulate insulin secretion *in vitro*, *Endocrine*, **3**, 55–59.
[7] Szecówka, J., Grill, V., Sandberg, E. and Efendic, S. (1982). Effect of GIP on the secretion of insulin and somatostatin and the accumulation of cyclic AMP *in vitro* in the rat, *Acta Endocrinol. Copenh.*, **99**, 416–421.
[8] Bonner-Weir, S. (1994). Regulation of pancreatic beta-cell mass *in vivo*, *Recent Prog. Horm. Res.*, **49**, 91–104.
[9] Brockenbrough, J. S., Weir, G. C. and Bonner-Weir, S. (1988). Discordance of exocrine and endocrine growth after 90% pancreatectomy in rats, *Diabetes*, **37**, 232–236.
[10] Dudley, D. T., Pang, L., Decker, S. J., Bridges, A. J. and Saltiel, A. R. (1995). A synthetic inhibitor of the mitogen-activated protein kinase cascade, *Proc. Natl. Acad. Sci. USA*, **92**, 7686–7689.
[11] Alessi, D. R., Cuenda, A., Cohen, P., Dudley, D. T. and Saltiel, A. R. (1995). PD 098059 is a specific inhibitor of the activation of mitogen-activated protein kinase kinase *in vitro* and *in vivo*, *J. Biol. Chem.*, **270**, 27489–27494.

[12] Hordijk, P. L., Verlaan, I., Van Corven, E. J. and Moolenaar, W. H. (1994). Protein tyrosine phosphorylation induced by lysophosphatidic acid in Rat-1 fibroblasts. Evidence that phosphorylation of map kinase is mediated by the $G_i$-p21ras pathway, *J. Biol. Chem.*, **269**, 645–651.

[13] Kahan, C., Seuwen, K., Meloche, S. and Pouysségur, J. (1992). Coordinate, biphasic activation of p44 mitogen-activated protein kinase and S6 kinase by growth factors in hamster fibroblasts. Evidence for thrombin-induced signals different from phosphoinositide turnover and adenylylcyclase inhibition, *J. Biol. Chem.*, **267**, 13369–13375.

[14] Quian, N. X., Winitz, S. and Johnson, G. L. (1993). Epitope-tagged $G_q$ alpha subunits: expression of GTPase-deficient alpha subunits persistently stimulates phosphatidylinositol-specific phospholipase C but not mitogen-activated protein kinase activity regulated by the M1 muscarinic acetylcholine receptor, *Proc. Natl. Acad. Sci. USA*, **90**, 4077–4081.

[15] Ohmichi, M., Koike, K., Nohara, A., Kanda, Y., Sakamoto, T., Zhang, Z. X., Hirota, K. and Miyake, A. (1994). Dopamine inhibits TRH-induced MAP kinase activation in dispersed rat anterior pituitary cells, *Biochem. Biophys. Res. Commun.*, **201**, 642–648.

[16] Pang, L., Decker, S. J. and Saltiel, A. R. (1993). Bombesin and epidermal growth factor stimulate the mitogen-activated protein kinase through different pathways in Swiss 3T3 cells, *Biochem. J.*, **289**, 283–287.

[17] Wang, Y., Simonson, M. S., Pouysségur, J. and Dunn, M. J. (1992). Endothelin rapidly stimulates mitogen-activated protein kinase activity in rat mesangial cells, *Biochem. J.*, **287**, 589–594.

[18] Asfari, M., Janjic, D., Meda, P., Li, G., Halban, P. A. and Wollheim, C. B. (1992). Establishment of 2-mercaptoethanol-dependent differentiated insulin-secreting cell lines, *Endocrinology*, **130**, 167–178.

[19] Kuo, W. N., Hods, D. and Kuo, F. N. (1973). Adenylate cyclase in islets of Langerhans. *J. Biol. Chem.*, **248**, 2705–2711.

[20] Verspohl, E. J. and Ammon, H. P. T. (1980). Evidence for the presence of insulin receptors in rat islets of Langerhans. *J. Clin. Invest.*, **65**, 1230–1237.

[21] Waga, I., Kume, K., Ferby, I., Honda, Z. and Shimizu, T. (1996). Micro-trap phosphorylation assay of nitrogen-activated protein (MAP) kinases to detect their activation by lipopolysaccharides, *J. Immunol. Methods*, **190**, 71–77.

[22] Akiyama, T., Ishida, J., Nakagawa, S., Ogawara, H., Watanabe, S., Itoh, N., Shibuya, M. and Fukami, Y. (1987). Genistein, a specific inhibitor of tyrosine-specific protein kinases, *J. Biol. Chem.*, **262**, 5592–5595.

[23] Verspohl, E. J., Tollkühn, B. and Kloss, H. (1995). Role of tyrosine kinase in insulin release in an insulin secreting cell line (INS-1), *Cell. Signal.*, **7**, 505–512.

[24] Hansson, A. (1991). Protein kinase C-dependent activation or a myelin basic protein kinase by gastrin-releasing peptide in Swiss 3T3 fibroblasts, *Cell. Signal.*, **3**, 293–298.

[25] Agostinis, P., Van Lint, J., Sarno, S., De Witte, P., Vandenheede, J. R. and Merlevede, W. (1992). Rapid stimulation of Ser/Thr protein kinases following treatment of Swiss 3T3 cells with bombesin. Involvement of casein kinase-2 in the signaling pathway of bombesin, *J. Biol. Chem.*, **267**, 9732–9737.

[26] Ohmichi, M., Koike, K., Nohara, A., Kanda, Y., Sakamoto, Y., Zhang, Z. X., Hirota, K. and Miyake, A. (1995). Oxytocin stimulates mitogen-activated protein kinase activity in cultured human puerperal uterine myometrial cells, *Endocrinology*, **136**, 2082–2087.

[27] Nohara, A., Ohmichi, M., Koike, K., Masumoto, N., Kobayashi, M., Akahane, M., Ikegami, H., Hirota, K., Miyake, A. and Murata, Y. (1996). The role of mitogen-activated protein kinase in oxytocin-induced contraction of uterine smooth muscle in pregnant rat, *Biochem. Biophys. Res. Commun.*, **229**, 938–944.

[28] Kubota, A., Yamada, Y., Yasuda, K., Someya, Y., Ihara, Y., Kagimoto, S., Watanabe, R., Kuroe, A., Ishida, H. and Seino, Y. (1997). Gastric inhibitory polypeptide activates MAP kinase through the wortmannin-sensitive and -insensitive pathways, *Biochem. Biophys. Res. Commun.*, **235**, 171–175.

[29] Hansson, A. (1994). Map kinase activation in Swiss 3T3 cells stimulated with gastrin-releasing peptide is associated with increased phosphorylation of a 78,000 M(r) protein immunoprecipitated by anti-raf kinase antiserum, *Cell. Signal.*, **6**, 423–431.

[30] Faure, M., Voyno-Yasenetskaya, T. A. and Bourne, H. R. (1994). cAMP and beta gamma subunits of heterotrimeric G proteins stimulate the mitogen-activated protein kinase pathway in COS-7 cells, *J. Biol. Chem.*, **269**, 7851–7854.

[31] Charles, H. C., Sun, H., Lau, L. F. and Tonks, N. K. (1993). The growth factor-inducible immediate-early gene 3CH134 encodes a protein-tyrosine-phosphatase, *Proc. Natl. Acad. Sci. USA*, **90**, 5292–5296.

[32] Porfiri, E. and McCormick, F. (1996). Regulation of epidermal growth factor receptor signaling by phosphorylation of the ras exchange factor hSOS1, *J. Biol. Chem.*, **271**, 5871–5877.

[33] Ptasznik, A., Traynor-Kaplan, A. and Bokoch, G. M. (1995). G protein-coupled chemoattractant receptors regulate Lyn tyrosine kinase. Shc adapter protein signaling complexes [published erratum appears in *J. Biol. Chem.* Oct. 13; 270(41), 24622], *J. Biol. Chem.*, **270**, 19969–19973.

[34] Wan, Y., Kurosaki, T. and Huang, X. Y. (1996). Tyrosine kinases in activation of the MAP kinase cascade by G-protein-coupled receptors, *Nature*, **380**, 541–544.

[35] Lev, S., Moreno, H., Martinez, R., Canoll, P., Peles, E., Musacchio, J. M., Plowman, G. D., Rudy, B. and Schlessinger, J. (1995). Protein tyrosine kinase PYK2 involved in $Ca^{2+}$-induced regulation of ion channel and MAP kinase functions, *Nature*, **376**, 737–745.

[36] Dikic, I., Tokiwa, G., Lev, S., Courtneidge, S. A. and Schlessinger, J. (1996). A role for Pyk2 and Src in linking G-protein-coupled receptors with MAP kinase activation, *Nature*, **383**, 547–550.

[37] Della-Rocca, G. J., Van Biesen, T., Daaka, Y., Luttrell, D. K., Luttrell, L. M. and Lefkowitz, R. J. (1997). Ras-dependent mitogen-activated protein kinase activation by G protein-coupled receptors. Convergence of $G_i$- and $G_q$-mediated pathways on calcium/calmodulin, Pyk2, and Src kinase, *J. Biol. Chem.*, **272**, 19125–19132.

[38] Aharonovitz, O., Aboulafia-Etzion, S., Leor, J., Battler, A. and Granot, Y. (1998). Stimulation of 42/44 kDa mitogen-activated protein kinases by arginine vasopressin in rat cardiomyocytes, *Biochim. Biophys. Acta*, **1401**, 105–111.

[39] Straub, S. G. and Sharp, G. W. (1996). Glucose-dependent insulinotropic polypeptide stimulates insulin secretion *via* increased cyclic AMP and $[Ca^{2+}]$ and a wortmannin-sensitive signalling pathway, *J. Biol. Chem.*, **271**, 1660–1668.

[40] Straub, S. G. and Sharp, G. W. (1996). A wortmannin-sensitive signal transduction pathway is involved in the stimulation of insulin release by vasoactive intestinal polypeptide and pituitary adenylate cyclase-activating polypeptide, *Biochem. Biophys. Res. Commun.*, **224**, 369–374.

[41] Gregersen, S. and Ahrén, B. (1996). Studies on the mechanisms by which gastrin releasing peptide potentiates glucose-induced insulin secretion from mouse islets, *Pancreas*, **12**, 48–57.
[42] Kubota, A., Yamada, Y., Yasuda, K., Someya, Y., Ihara, Y., Kagimoto, S., Watanabe, R., Kuroe, A., Ishida, H. and Seino, Y. (1997). *Biochem. Biophys. Res. Commun.*, **235**, 171–175.
[43] Strakova, Z., Copland, J. A., Lolait, S. J. and Soloff, M. S. (1998). ERK2 mediates oxytocin-stimulated PGE2 synthesis, *Am. J. Physiol.*, **274**, E634–641.
[44] Romanelli, A. and Van de Werve, G. (1997). Activation of mitogen-activated protein kinase in freshly isolated rat hepatocytes by both a calcium- and a protein kinase C-dependent pathway, *Metabolism*, **46**, 548–555.
[45] Kribben, A., Wieder, E. D., Li, X., Van Putten, V., Granot, Y., Schrier, R. W. and Nemenoff, R. A. (1993). AVP-induced activation of MAP kinase in vascular smooth muscle cells is mediated through protein kinase C, *Am. J. Physiol.*, **265**, C939–945.
[46] Nishioka, N., Hirai, S., Mizuno, K., Osada, S., Suzuki, A., Kosaka, K. and Ohno, S. (1995). Wortmannin inhibits the activation of MAP kinase following vasopressin V1 receptor stimulation, *FEBS Lett.*, **377**, 393–398.
[47] Díaz-Meco, M. T., Lozano, J., Municio, M. M., Berra, E., Frutos, S., Sanz, L. and Moscat, J. (1994). Evidence for the *in vitro* and *in vivo* interaction of Ras with protein kinase C zeta, *J. Biol. Chem.*, **269**, 31706–31710.
[48] Liao, D. F., Monia, B., Dean, N. and Berk, B. C. (1997). Protein kinase C-zeta mediates angiotensin II activation of ERK1/2 in vascular smooth muscle cells, *J. Biol. Chem.*, **272**, 6146–6150.
[49] Hellerström, C. and Swenne, I., Growth pattern of pancreatic islets in animals, In: The Diabetic Pancreas Volk, B. W. and Arquilla, E. R. (Eds.) Plenum, New York, 1985, pp. 53–79.
[50] Bonner-Weir, S. and Smith, F. E. (1994). Islet cell growth and the growth factors involved, *Trends Endocrinol. Metab.*, **5**, 60–64.
[51] Huotari, M. A., Palgi, J. and Otonkoski, T. (1998). Growth factor-mediated proliferation and differentiation of insulin-producing INS-1 and RINm5F cells: identification of betacellulin as a novel beta-cell mitogen, *Endocrinology*, **139**, 1494–1499.
[52] Bold, R. J., Lowry, P. S., Oshozuka, J., Battey, J. F., Townsend, C. M. and Thompson, J. C. (1994). Bombesin stimulates the *in vitro* growth of human gastric cancer cell line, *J. Cell. Physiol.*, **161**, 519–525.
[53] Hajri, A., Koenig, M., Garaud, J. C. and Damgé, C. (1992). Gastrin-releasing peptide: *in vivo* and *in vitro* growth effects on an acinar pancreatic carcinoma, *Cancer Res.*, **52**, 3726–3732.
[54] Alexander, R. W., Upp, J. R. Jr., Poston, G. J., Townsend, C. M., Singh, P. and Thompson, J. C. (1988). Bombesin inhibits growth of human pancreatic adenocarcinoma in nude mice, *Pancreas*, **3**, 297–302.
[55] Persaud, S. J., Wheeler-Jones, C. P. and Jones, P. M. (1995). The mitogen-activated protein kinase cascade in rat islets of Langerhans. *Biochem. Soc. Trans.*, **23**, 221S.

